# Cancer of the Tongue and Tobacco

**Published:** 1919-01-18

**Authors:** 


### CANCER OF THE TONGUE AND TOBACCO.

Among the subordinate questions relating to cancer there has appeared every now and again the inquiry whether the disease is or is not an increasing quantity. Taking the official figures of the causes of death the answer to this doubt is decidedly in the affirmative, and many clinical observers, including so experienced a surgeon as the late Sir Jonathan Hutchinson, have expressed a confident conclusion in the same direction. Yet it must be admitted that there has always been some uncertainty whether what appears to be an obvious increase may not perhaps be explained by improved methods of diagnosis.

By microscopic and other methods malignancy is now recognised in conditions where some years ago it would not have been suspected. Hence whether the increase in the deaths attributed to cancer is a real increase or only an apparent one is a question which admits some difference of opinion. The balance is certainly towards the affirmative and this view is strongly favoured by the facts and figures submitted by Mr. D'Arcy Power in his recent Bradshaw lecture on Cancer of the Tongue. According to Mr. Power the disease is hardly mentioned by surgical writers prior to the seventeenth century, and the first recorded case occurred in 1634. What a contrast such a statement presents to present-day experience our professional readers will at once appreciate, and the figures supplied by the Superintendent of Statistics at Somerset House will make the position apparent to all. Writing in 1909 the Superintendent said "the recorded mortality has increased among males by no less than 228 per cent. in forty-one years." Here at all events it is impossible to believe that we are dealing with a situation which is a mere expression of more exact diagnosis.

Searching for a cause for this increase of lingual cancer Mr. Power finds that the increase is not associated with any hereditary tendency to carcinoma, and he does not believe that specific disease has more than a predisposing influence. It is necessary, therefore, to find an exciting cause, and this must be an agency which acts on men much more than on women and has become increasingly operative during the last fifty years. Dental caries and pyorrhœa alveolaris can be dismissed as not satisfying either of these two conditions. On the other hand, a strong case can be presented against tobacco. Its use has enormously extended during the past forty years and, at all events until quite recently, the habit was cultivated chiefly by men and not by women. There is a consensus of opinion that local irritation is a large element in the determination of the site of cancer, and tobacco smoking may supply this, partly in the shape of heat and partly in the shape of nicotin. With a predisposition to cancer of the tongue established by specific disease and with an extension of the tobacco habit in both sexes Mr. Power anticipates a still further increase in lingual carcinoma for, so he affirms, specific disease is now more prevalent in the younger generation than it has been for some years, and the cigarette numbers its devotees in wider and wider circles and claims the gentler as well as the sterner sex.

